# Genetic Diversity and Core Germplasm Development in Yunnan Tartary Buckwheat Based on Phenotypic and SNP Data

**DOI:** 10.3390/plants15081197

**Published:** 2026-04-14

**Authors:** Bingxin Zhai, Daowang Sun, Chunyan Huang, Zihan Zhao, Jiaxing Xie, Xin Liu, Wenjie Lu, Guang Wang, Lihua Wang

**Affiliations:** Biotechnology and Germplasm Resources Institute, Yunnan Academy of Agricultural Sciences (Yunnan Provincial Key Laboratory of Agricultural Biotechnology/Key Laboratory of Southwestern Crop Gene Resources and Germplasm Innovation/Ministry of Agriculture and Rural Affairs), Kunming 650205, China; xiaozhai943@163.com (B.Z.); dwsun03@sina.com (D.S.); hcy456678@163.com (C.H.); 17513720467@163.com (Z.Z.); 18053241733@163.com (J.X.); 17790631569@163.com (X.L.); luwenjie1976@163.com (W.L.)

**Keywords:** Tartary buckwheat, whole-genome resequencing, SNP, genetic diversity, population structure, core germplasm

## Abstract

Southwest China harbors the world’s richest germplasm resources of Tartary buckwheat (*Fagopyrum tataricum*). However, their effective utilization is severely constrained by poor management and narrow genetic diversity. Developing a core collection is a key strategy for overcoming these bottlenecks and facilitates the efficient conservation and utilization of germplasm resources. Therefore, we aimed to assess the genetic diversity and population structure of a Tartary buckwheat germplasm collection from Yunnan Province and adjacent regions to establish a core collection. Whole-genome resequencing and phenotyping of four key agronomic traits were performed on 313 Tartary buckwheat accessions. Population genetic structure, differentiation, and diversity parameters were analyzed using single-nucleotide polymorphism (SNP) data. We obtained 3,433,676 high-quality SNP markers. The 313 accessions were classified into four ancestral populations and three phylogenetic groups, which revealed the complex patterns of genetic differentiation and gene flow. Phenotypic traits exhibited high genetic diversity, with wide variation ranges in key agronomic traits such as plant height, stem diameter, and branching characteristics, highlighting the richness of the germplasm resources. By integrating the phenotypic and SNP data, we constructed a core collection (Core-Merge) comprising 105 accessions (33.55% of the original collection). Core-Merge showed no significant differences in phenotypic traits compared to the original collection, exhibited a similar distribution in principal coordinate analysis, and demonstrated low kinship among individuals. The collection established in this study, Core-Merge, captures the maximum phenotypic and genotypic variability present in the original germplasm. The core germplasm collection provides a valuable foundation for the efficient conservation of, genetic research on, and molecular breeding of Tartary buckwheat.

## 1. Introduction

*Fagopyrum tataricum* Gaertn., commonly referred to as Tartary buckwheat, is indigenous to the cold, elevated regions of Southwest China, including the Yunnan–Guizhou and Qinghai-Tibet plateaus [1,2,3]. Tartary buckwheat is an important crop for both dietary and medicinal applications, as it is rich in unique bioactive compounds such as rutin and D-chiro-inositol. Tartary buckwheat also contains high-quality proteins, dietary fiber, vitamins, and essential trace elements [4]. These components are closely associated with various physiological benefits, such as hypoglycemic, hypolipidemic, and antihypertensive effects [5,6]. Tartary buckwheat is also notable for its ability to withstand cold temperatures, thrive in poor soil conditions, and complete a brief growth cycle, making it an essential crop for farmers in mountainous areas [7,8]. Southwest China is the native home of Tartary buckwheat and contains the world’s richest genetic resources of this crop [9]. The Tartary buckwheat processing and food industry encompasses companies that produce primary products such as flour, health foods, and functional ingredients. However, the development of the Tartary buckwheat processing and food industry is severely constrained by critical bottlenecks in germplasm management, including inadequate documentation, disorganized collection, and inappropriate curation. In addition, progress is further hindered by limitations in breeding applications, such as the scarcity of elite lines and low utilization rates. Therefore, it is essential to implement systematic collection, precise identification, and comprehensive multi-trait evaluation practices to clarify the germplasm inventory and establish a high-quality core collection.

This need is partly due to redundancy and overlap within collections, as many accessions are traditional landraces or derivatives from conventional breeding techniques that often share limited genetic backgrounds [10]. While the quantity of collected resources is substantial, their effective deployment in breeding has been insufficient. Significant global efforts have been made to collect Tartary buckwheat germplasms [10], resulting in the accumulation of numerous accessions with rich diversity in terms of agronomic traits, stress resistance [11], nutritional quality [12,13], and ecological adaptation [14]. These efforts have involved systematic field surveys, in italic conservation in gene banks, and phenotypic evaluation programs. However, despite the scale of these collections, the management and preservation of these extensive resources remain a significant challenge, one hindered by genetic impurity, inconsistent performance, and a lack of systematic characterization. A core collection serves as a key means of overcoming these bottlenecks by concentrating genetic diversity into a smaller, representative set, thereby enhancing the efficiency of genetic research and breeding [15]. Several research groups have developed core collections across different crops. Song et al. developed a core collection of 172 maize accessions using phenotypic traits and single-nucleotide polymorphism (SNP) markers [16]. Yang et al. screened a core collection of 30 garlic cultivars based on genetic diversity and population structure [17]. Tian et al. constructed a core germplasm bank of 21 cold-resistant rubber tree accessions based on SNP-based genetic diversity analysis [18]. Cheng et al. established a core collection of 165 Tartary buckwheat accessions based on an analysis of genetic diversity across Chinese germplasms [15]. The southwest region of China, as the crop’s native home, should develop its own core collection given its abundant resources and the closer genetic relationships among its accessions. In addition, a core collection can significantly enhance the efficiency of genotype–phenotype association studies, facilitate the discovery of genes governing important agronomic traits, and accelerate molecular marker development, as demonstrated in regard to tomato [19] and apple [20]. These affordances, in turn, support targeted breeding for yield, quality, and stress tolerance. Ultimately, a representative core collection serves as an indispensable platform for advancing genetic research and enabling modern, precise breeding strategies. Achieving this end is essential in order to overcome the longstanding limitations in Tartary buckwheat improvement and meet the corresponding growing industrial demand.

To use genetic resources more effectively, it is essential to move beyond mere collection and conduct systematic analyses of genetic diversity and population structure. Various molecular markers have been employed to assess genetic relationships and variation among accessions, including amplified-fragment-length polymorphism (AFLP), random amplified polymorphic DNA (RAPD), simple sequence repeat (SSR), insertion–deletion (InDel), and SNP markers [7,15,21,22,23,24]. SSR and AFLP markers were widely used in early studies, while recent advances in high-throughput sequencing have enabled the development of genome-wide SNP markers with higher resolution and reproducibility. Compared with traditional molecular markers, SNP markers are more abundant, evenly distributed across the genome, and suitable for high-throughput genotyping, making them particularly powerful for population structure analysis, genetic diversity assessment, and core collection construction.

Whole-genome resequencing has emerged as a powerful tool for large-scale genetic diversity analysis and germplasm characterization, enabling the identification of millions of SNPs across the entire genome with high resolution. Reference genomes underpin functional genomics and provide the foundation for genome-wide marker development. In regard to Tartary buckwheat, there has been significant progress in genomic resource development in recent years. The genome of *F. tataricum* has been sequenced and assembled, with reported genome sizes of approximately 489.3 Mb. The reference genome (GCA_002319775.1) provides a critical reference framework for genome-wide marker development [25]. Building on this reference genome, researchers have identified several genomic loci that contribute to the formation of important quality and yield traits of Tartary buckwheat [26]. Earlier molecular marker studies on Tartary buckwheat, employing SNP [10], AFLP [21], SSR [27,28], and RAPD [29] markers, revealed considerable genetic variation among accessions from different geographic origins, laying the groundwork for germplasm evaluation. More recently, genome-wide SNP genotyping was applied to investigate population structure, domestication history, and adaptive evolution regarding *F. tataricum*, demonstrating the power of high-density markers with respect to resolving fine-scale genetic differentiation [10]. These genomic resources and prior findings provide both the technical foundation and biological context for the SNP-based approach adopted in this study.

Although previous studies have characterized the genetic diversity and population structure of Tartary buckwheat at the national scale, there is still a lack of a dedicated core collection for Yunnan germplasm, which represents the richest genetic reservoir of this crop. Currently, integrating high-density SNPs markers with comprehensive phenotypic evaluation serves as a precise approach to capturing the maximum genetic and trait variability present in diverse germplasm pools. This approach has been successfully demonstrated in regard to crops, such as maize [16] and tea [30]. Notably, previous studies have shown that core collections constructed by integrating genetic and phenotypic data effectively preserve traits and improve genetic diversity [26,27]. The establishment of a scientifically curated core collection, based on such multidimensional data, can represent the broad diversity of original resources with a minimal, manageable set of accessions, thereby providing a streamlined and efficient resource for downstream applications. However, there are no reports detailing the construction of a core collection integrating both phenotypic and genetic data for Tartary buckwheat, making such an approach essential for advancing research on this plant.

Therefore, the purposes of this study were (1) to assess the genetic diversity and population structure of Yunnan Tartary buckwheat germplasm resources and (2) establish a core collection for this material based on both phenotypic and SNP data.

## 2. Results

### 2.1. Whole-Genome Resequencing Results

Whole-genome resequencing of 313 Tartary buckwheat was performed using the MGI T7 platform. As reported in Supplementary Table S1, we obtained a total of 3303.83 Gb of clean data, with an average of 10.56 Gb of clean data per accession. The average Q20 and Q30 values were 98.43% and 95.33%, respectively. The GC content ranged from 39.00% to 41.62%, indicating high library quality and accurate, reliable sequencing results that are suitable for subsequent SNP marker discovery. The average sequencing depth reached approximately 22.98× (Supplementary Table S2). The reads were aligned to the Tartary buckwheat reference genome (GCA_002319775.1) [25] using Sentieon software(v202212.01), and the percentage of reads mapped to the reference genome ranged from 97.62% to 99.40% (Supplementary Table S2). These results provide a reliable data foundation for subsequent SNP marker discovery and genetic analysis.

### 2.2. SNP Identification

**Figure 1:**
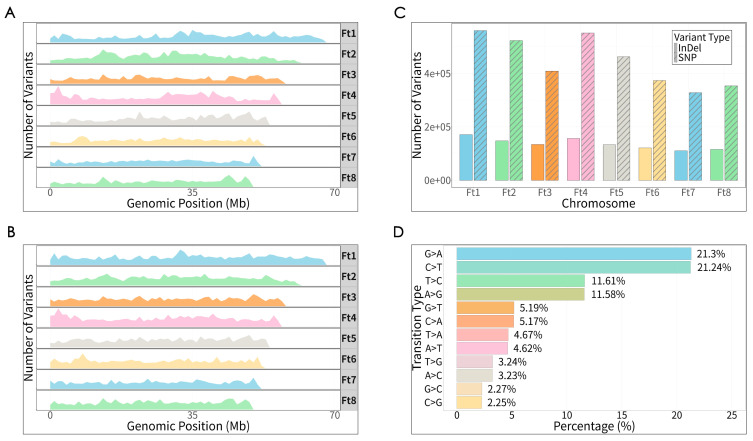
SNP and InDel screening and in silico simulation: (**A**) distribution of SNPs; (**B**) distribution of InDels; (**C**) percentages of various transition rates; (**D**) transition frequency distribution.

A total of 3,433,676 SNPs and 130,128 InDels were identified in the 313 Tartary buckwheat accession. The quantities of SNPs on chromosomes 1 to 8 were 541,044, 503,451, 395,222, 530,242, 446,156, 360,460, 316,420, and 340,681, respectively. A total of 19,763, 20,109, 13,909, 21,601, 16,513, 13,297, 11,807, and 13,129 InDels were detected on chromosomes 1 to 8, respectively. The SNPs and InDels were unevenly distributed across the eight chromosomes of Tartary buckwheat, with higher numbers observed on chromosomes 1, 2, and 4 and the fewest observed on chromosome 7 (Figure 1A,B). The overall average SNP and InDel densities were 7.564 and 0.287 per kb, respectively. Chromosome 4 exhibited the highest SNP frequency (9.359 SNPs per kb) and the highest InDel frequency (0.381 InDels per kb (Supplementary Table S3)). Chromosome 7 showed the lowest SNP frequency (6.139 SNPs per kb) and the lowest InDel frequency (0.229 InDels per kb). At the genome-wide level, the percentages of SNPs associated with transitions and proportions were 68.22% and 31.78%, respectively, resulting in a transition-to-transversion (Ts/Tv) ratio of 2.146. The transition frequencies of C/T and G/A were 21.24% and 21.30%, respectively, while the transversion frequencies of T/C, A/G, G/T, and A/C were 11.61%, 11.58%, 5.19%, and 5.17%, respectively (Figure 1C, Supplementary Table S4). After filtering, a total of 1,114,360 SNPs were obtained for subsequent analysis. These results confirmed that the sequencing data were reliable and could be used for subsequent analysis.

### 2.3. Genetic Diversity Analysis

Based on the filtered SNP data, we calculated multiple genetic diversity parameters, including observed heterozygosity (Ho), expected heterozygosity (H_E_), nucleotide diversity (*π*), major allele frequency (MAF), the inbreeding coefficient (FIS), and polymorphism information content (PIC). The number of SNPs across populations ranged from 1,036,296 to 1,113,622, with an average of 1,092,159 (Table 1). Ho values ranged from 0.2896 to 0.2982, with a mean of 0.2935. H_E_ values varied from 0.3029 to 0.3277, averaging 0.3175. π ranged from 3.0699 × 10^−3^ to 3.2973 × 10^−3^, with a mean of 3.1984 × 10^−3^. MAF values ranged from 0.2268 to 0.2476, averaging 0.2387. FIS showed the widest relative variation, ranging from 0.0372 to 0.0907, with a mean of 0.0753. PIC values ranged from 0.2515 to 0.2696, averaging 0.2595. The DXB population showed the highest values for the number of SNPs (1,113,622), H_E_ (0.3277), Ho (0.2982), π (3.2973 × 10^−3^), and MAF (0.2476), while the DX population displayed the highest PIC (0.2696) and FIS (0.0907) values. Conversely, the DDN population showed relatively lower genetic diversity, with the lowest values for H_E_ (0.3029), π (3.0699 × 10^−3^), MAF (0.2268), FIS (0.0372), and PIC (0.2515). Overall, these results indicate that the DXB and DX populations exhibit the highest genetic diversity among all the populations, while DDN shows the lowest diversity.

### 2.4. Population Genetic Structure

**Figure 2:**
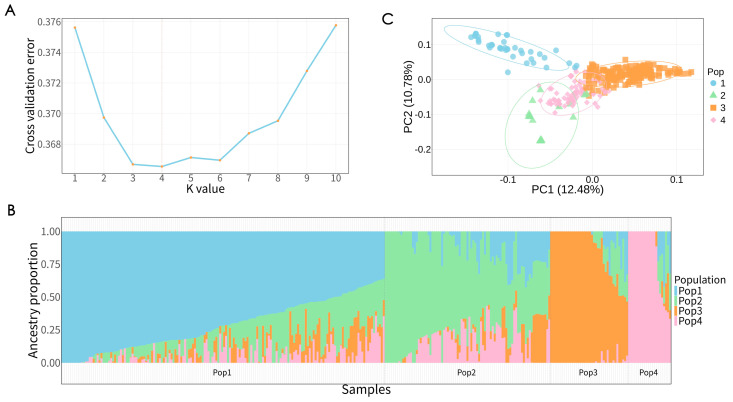
Population genetic structure: (**A**) Cross-validation errors. (**B**) Ancestral component analysis at K = 4. Each accession is represented as a vertical line divided into colored segments, reflecting the estimated membership proportions in each cluster. (**C**) Principal component analysis. Pop1, ancestral group 1; Pop2, ancestral group 2; Pop3, ancestral group 3; Pop4, ancestral group 4.

By evaluating the dynamic changes in population structure under different K values (K = 2 to 10), we analyzed the genetic structure of the germplasm accessions. As shown in Figure 2A, the cross-validation (CV) error value was lowest at K = 4, indicating that the optimal population grouping corresponded to four ancestral populations. The first ancestral group, group 1 (Pop1), consisted of 166 accessions, comprising DXB (62), DDB (31), DZ (27), YJZY (23), DX (11), DXN (7), and DDN (5) (Figure 2B, Supplementary Table S5). Group 2 (Pop2) contained 85 accessions, primarily associated with DXN and DDN. Group 3 (Pop3) contained 40 accessions, with YJZY and DDB predominantly associated with this cluster. Group 4 (Pop4) comprised 22 accessions, mainly represented by DDN. The third ancestral group (Pop3) consisted of 166 accessions, comprising DXB (62), DDB (31), DZ (27), YJZY (23), DX (11), DXN (7), and DDN (5). The fourth ancestral group (Pop4) contained 85 accessions, primarily associated with DXN and DDN. The results of the principal component analysis (PCA) (Figure 2C) reveal a distinct structure among populations along PC1 indicating clear differentiation. Furthermore, on PC2, the intra-population dispersion depicted by the ellipses offers insight into the variability within each population. The three principal components, PC1, PC2, and PC3, account for 12.48%, 10.78%, and 7.22% of the total genetic variance, respectively.

**Figure 3:**
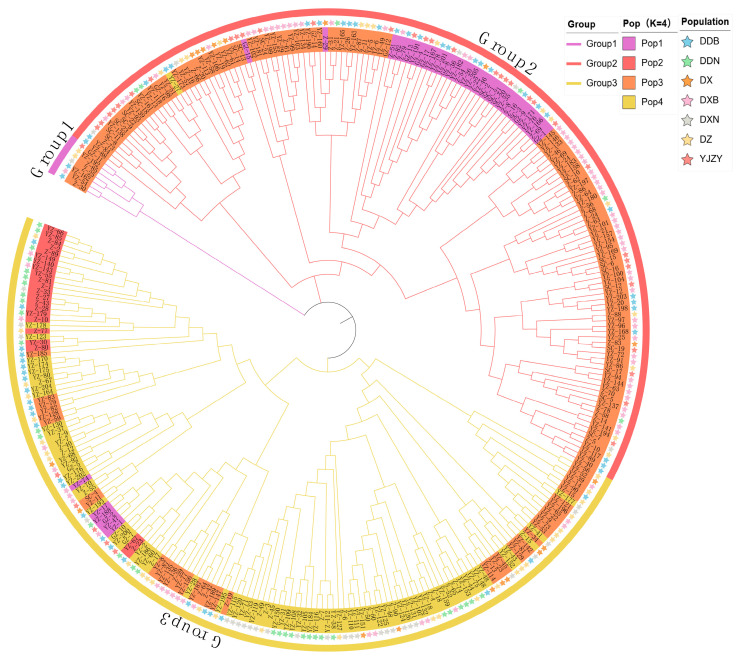
Neighbor-joining (NJ) phylogenetic tree of 313 Tartary buckwheat accessions constructed using SNP data. Here, “group” was used for constructing the NJ phylogenetic tree, “pop” was used for constructing the population structure, and “population” represents the origin of the germplasm resources.

A total of 313 accessions were analyzed using a neighbor-joining (NJ) phylogenetic tree to elucidate the relationships among the inferred populations, which were categorized into three distinct groups (Figure 3). Group 1 contained seven accessions (2.22%), including DDB (2), DZ (2), DXB (1), DXN (1), and YJZY (1). Group 2 contained 152 accessions (48.58%), of which the DXB, YJZY, and DDB populations had 55, 33, and 30 accessions, respectively. Group 3, comprising 154 accessions (49.20%), consisted of seven populations: DDN (32), DZ (30), DXN (27), DDB (26), DXB (24), DX (10), and YJZY (5). Notably, when examining the distribution of population codes (DDB, DDN, DX, DXB, DXN, DZ, and YJZY) within each phylogenetic group, we found that accessions originating from the same sub-regional population (e.g., DXB and DDB) showed a tendency to co-occur within the same group. This pattern suggests that geographic proximity within Yunnan Province influences genetic similarity and population clustering, consistent with isolation-by-distance effects.

Genetic differentiation among the seven populations was assessed using Fst and Nm analyses (Table 2). The Fst values across all population pairs ranged from −0.0005 to 0.0456, while Nm values ranged from −476.69 to 49.91. A negative Fst value (−0.0005) was observed between the DX and DZ populations, indicating almost no genetic differentiation and a very high degree of genetic similarity between these two populations. The highest Fst value (0.0456) was found between the YJZY and DDN populations, followed by DXB and DDN (0.0372) and YJZY and DXN (0.0362), with corresponding Nm values of 5.23, 6.48, and 6.64, respectively. These results suggest a certain degree of genetic differentiation among these populations. Excluding the negative value, the lowest Fst value (0.0050) was recorded between DDB and DZ, followed by DDB and YJZY (0.0066) and DX and DXB (0.0069). These population pairs exhibited high levels of gene flow, with Nm values of 49.91, 24.35, and 6.65, respectively. These findings reveal a complex genetic structure and differentiated gene flow patterns among the populations, providing a genetic basis for the future conservation and utilization of germplasm resources.

### 2.5. Phenotypic Diversity

To understand the variation in phenotypic traits among different individuals, statistical analysis was performed on the phenotypic traits (BNMS, NNMS, SD, and PH) of the 313 Tartary buckwheat accessions (Supplementary Table S6). Among the four phenotypic traits, BNMS averaged 6.8, NNMS averaged 19.1, SD averaged 9.8 mm, and PH averaged 151.3 cm (Table 3). The Shannon’s genetic diversity index (H′) values for the four phenotypic traits ranged from 0.8 to 1.8, with an average of 1.48, indicating a high level of genetic diversity in these traits. The coefficient of variation (CV%) ranged from 8.1% to 21.3%, with an average of 12.01%, suggesting considerable variability in phenotypic traits across different accessions. The wide ranges observed for key traits further highlight the richness of the germplasm. For example, PH ranged from 121.8 cm to 209.4 cm, and SD varied from 7.6 mm to 13.1 mm, indicating the presence of both compact and tall growth types as well as structural diversity. Similarly, BNMS exhibited substantial variation (4.0–24.3), reflecting diverse plant architectures among accessions. These outstanding phenotypic differences provide valuable resources for breeding programs targeting yield potential, lodging resistance, and environmental adaptability.

**Figure 4:**
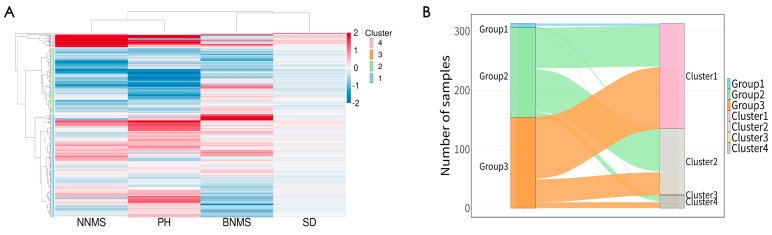
Clustering and group distribution of Tartary buckwheat individuals based on phenotypic and phylogenetic analyses: (**A**) heatmap of the phenotypic trait levels showing the clustering of 313 Tartary buckwheat accessions, and (**B**) Sankey diagram showing the distribution flow of four clusters of phenotypic-trait-based clustering into three groups of SNPs in a neighbor-joining phylogenetic tree. BNMS, number of branches on the main stem; NNMS, number of nodes on the main stem; SD, stem diameter; PH, plant height.

Using hierarchical clustering of phenotypic data, we grouped the 313 accessions into four major clusters of 178, 112, and 22 germplasms and 1 germplasm (Figure 4A). Next, a Sankey diagram was constructed to compare the groupings from phenotypic- trait-based clustering (four clusters) with those from the SNP neighbor-joining phylogenetic tree (three groups). The phylogenetic tree and phenotypic-trait-based clustering were not fully aligned (Figure 4B). Group 1 was predominantly assigned to Cluster 1 (57.14%, *n* = 4), followed by Cluster 2 (28.57%, *n* = 2) and Cluster 4 (14.29%, *n* = 1). Group 2 was distributed almost equally between Cluster 1 (45.39%, *n* = 69) and Cluster 2 (47.37%, *n* = 72), with a smaller proportion assigned to Cluster 4 (7.24%, *n* = 11). Group 3 was strongly associated with Cluster 1 (68.18%, n = 105), while a moderate proportion was classified into Cluster 2 (24.68%, *n* = 38). Only a minor share was found in Cluster 4 (6.49%, *n* = 10), and a single case (0.65%) was assigned to Cluster 3. As these data show, the clear differentiation in genetic and phenotypic traits across accessions indicates that the diversity within the core set cannot be fully captured by a single type of dataset.

### 2.6. Construction of Core Germplasm Collection

To balance and maximize the capture of phenotypic and genotypic variability from the original collection, we constructed independent core germplasm subsets using both genotypic and phenotypic data derived from the 313 Tartary buckwheat germplasm accessions. The core subsets were generated using Core Hunter 3, which optimizes subset selection based on diversity criteria for both SNP and phenotypic datasets. At the 30% sampling ratio, the SNP-based subset demonstrated genetic diversity metrics (Ho, H_E_, PIC, and π) most similar to those of the original germplasm collection, and the π value was superior to that of the other candidates (Table 4). This finding indicates that a 30% sampling ratio, comprising 93 accessions (Core1), most effectively eliminated redundant germplasms while preserving the genetic diversity of the original collection (Supplementary Table S7). For the subset based on phenotypic traits, we selected a 5% construction proportion as Core2 (15), which achieved an MD of 0%, a VD of 56.25%, a VR of 133.75%, and a CR of 97.26% (Supplementary Tables S7 and S8).

**Figure 5:**
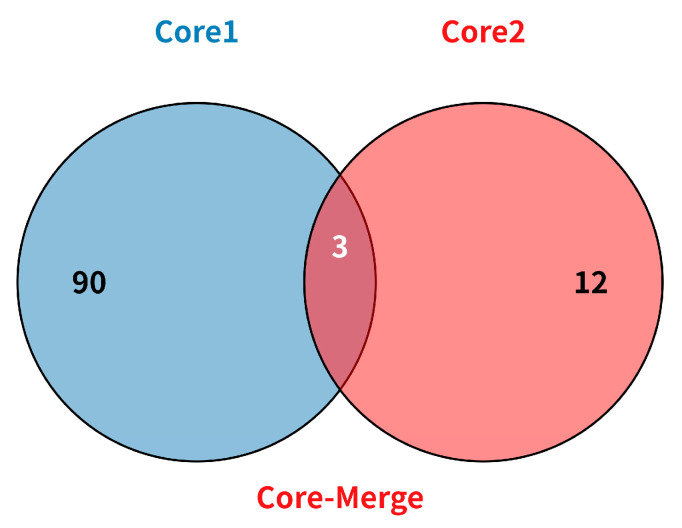
Merging Core1 and Core2 formed the core collection (Core-Merge).

**Figure 6:**
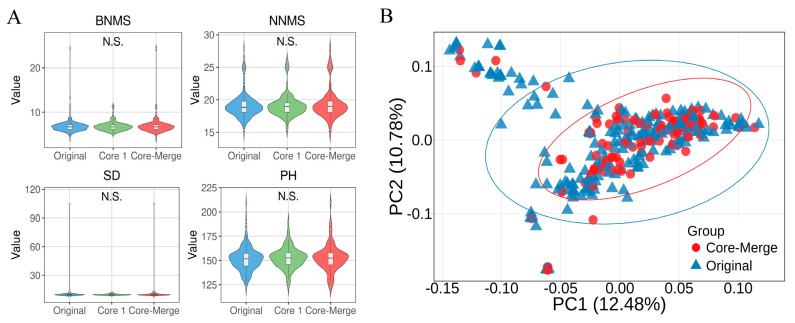
Comparison of distributions among the original, Core1, and Core-Merge datasets: (**A**) distribution of phenotypic trait values in the Core-Merge (*n* = 105), Core1 (*n* = 93), and original (*n* = 313) germplasms, and (**B**) principal coordinate analysis (PCoA) based on SNP marker genotypic data for the core germplasm (*n* = 105) and the original germplasm (*n* = 313). N.S. indicates no significant difference.

As shown in Figure 5 and Supplementary Table S7, Core1 and Core2 had 90 and 12 unique germplasms, respectively, and shared 3 germplasms. Merging Core1 (93) and Core2 (15) formed the core collection (Core-Merge), which comprises 105 germplasms representing 33.55% of the original collection. Compared to the Core1 germplasms, the Core-Merge germplasms exhibited extreme values for the traits BNMS, SD, and PH, suggesting that integrating phenotypic data helps capture additional phenotypic variation beyond what genetic data alone can achieve. Analysis of variance (ANOVA) confirmed there were no significant differences among the Core-Merge, Core1, and original germplasms with respect to all four traits (*p* > 0.05), validating the representativeness of the Core-Merge (Figure 6A). In the principal coordinate analysis (PCoA), the Core-Merge germplasms exhibited a distribution similar to that of the original germplasm (Figure 6B). These results demonstrate the successful development of Core-Merge, which captures the maximum phenotypic and genotypic variability present in the original set of 313 Tartary buckwheat accession germplasms.

### 2.7. Analysis of Kinship in Core-Merge

**Figure 7:**
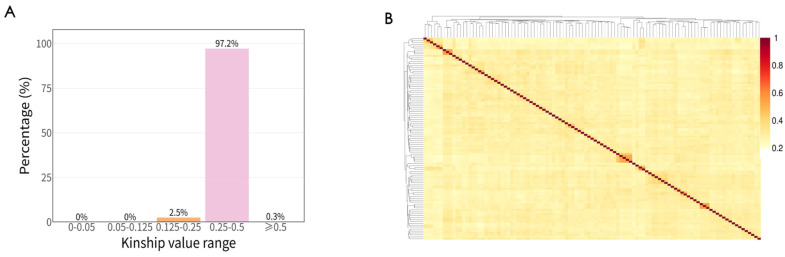
Analysis of phylogenetic relationships in the Core-Merge dataset: (**A**) histogram displaying the phylogenetic relationships of the Core-Merge collection of Tartary buckwheat, and (**B**) kinship matrix of the Core-Merge collection of Tartary buckwheat.

In an ideal core collection, kinship among individuals should be minimized to ensure that it adequately represents the genetic diversity of the entire germplasm. We performed kinship analysis to evaluate the genetic relationships within Core-Merge. The results revealed that 97.2% of the core germplasm accession pairs exhibited kinship values ranging from 0.25 to 0.5, while 2.5% had kinship values between 0.125 and 0.25. Only 15 pairs showed kinship values greater than 0.5 (Figure 7). Kinship values between 0.25 and 0.5 indicate a moderate level of genetic relationship, which is expected in a geographically concentrated germplasm collection from a single region. Nevertheless, the overall kinship distribution suggests that the Core-Merge collection avoids excessive redundancy and captures a broad range of the genetic diversity present in the original collection.

## 3. Discussion

Genetic diversity is the basis for crop breeding and germplasm resource conservation, holding significant importance for evaluating population genetic potential, guiding breeding strategies, and formulating effective conservation measures [31]. A core germplasm collection is designed to retain genetic diversity while reducing redundancy, thereby enhancing resource utilization efficiency, as demonstrated in crops such as winter wheat [32] and barley [33]. Therefore, this study provides a scientific basis for constructing a core germplasm collection by comprehensively assessing the genetic and phenotypic diversity of Tartary buckwheat. Yunnan Province boasts abundant and historically rich Tartary buckwheat germplasm resources. However, their effective conservation and utilization remain insufficient. In this study, we analyzed the genetic diversity of 313 Tartary buckwheat accessions belonging to six populations in Yunnan Province and the YJZY population from Sichuan and Guizhou Provinces using SNP data. The average values of Ho, H_E_, π, MAF, and PIC were 0.2935, 0.3175, 3.1984 × 10^−3^, 0.2387, and 0.2595, respectively. Previous studies identified 1,095,748 [26], 1,004,824 [12], and 1,103,732 [13] SNPs across 510, 200, and 226 Tartary buckwheat accessions, respectively. In this study, a total of 1,114,360 SNPs were obtained, with filtering, from 313 Tartary buckwheat accessions, a finding consistent with previous studies [12,13,26]. The Ho of Tartary buckwheat in Yunnan is higher than the average Ho of Chinese Tartary buckwheat, indicating relatively rich genetic diversity in this region, a result consistent with findings reported for other crops such as millet [34] and wheat [32].

Accurate evaluation of agronomic traits is essential for the effective utilization of favorable alleles and germplasm improvement [31]. In this study, the measured traits (BNMS, NNMS, SD, and PH) exhibited substantial variation, reflecting the rich genetic diversity within the population. These pronounced variations, particularly in PH, SD, and branching traits (BNMS and NNMS), highlight the presence of outstanding phenotypes within the germplasm, further demonstrating its richness and potential value for targeted breeding. Notably, populations such as DXB and DX showed higher levels of diversity, indicating their potential as valuable sources for selecting genotypes with enhanced adaptability [26,35]. Overall, this genetically diverse germplasm provides a solid foundation for both conventional and marker-assisted breeding aimed at trait improvement [36]. In addition to evaluating genetic diversity within Tartary buckwheat, it is important to compare these findings with those reported for other crop species. From a phenotypic perspective, the relatively high coefficients of variation (8.1–21.3%) and Shannon diversity index values (H′ = 0.8–1.8) observed in this study indicate substantial morphological variability among accessions. Similar patterns of phenotypic variation have been reported in regard to crops such as maize [16] and barley [33], where traits related to plant architecture show considerable diversity and contribute to environmental adaptation and yield improvement. From a molecular perspective, the genetic diversity parameters (H_E_ = 0.3175, π = 3.1984 × 10^−3^, and PIC = 0.2595) obtained in this study are comparable to those reported for self-pollinating crops such as barley [33], while the inconsistency observed between phenotypic clustering and SNP-based grouping has also been reported in crops such as rice [24]. Furthermore, the low Fst values observed among populations indicate weak genetic differentiation, which is consistent with findings from other crop germplasm studies. Notably, the inconsistency between phenotypic clustering and SNP-based grouping observed in this study has also been reported in regard to crops such as rice [24], where environmental effects influence phenotypic expression. These comparisons further support the importance of integrating phenotypic and molecular data to comprehensively evaluate germplasm diversity.

Beyond genetic diversity, analysis of population structure revealed fascinating insights. Understanding population structure is crucial for constructing representative core germplasm resources [36]. In this study, we analyzed the population structure of Tartary buckwheat germplasm resources using both SNP and phenotypic data. Based on SNP data, we divided 313 Tartary buckwheat accessions into four distinct groups via population structure analysis and PCA (Figure 2). An NJ phylogenetic tree further classified the germplasm into three distinct groups, which largely corresponded to their geographical origins. This trend aligns well with findings regarding Tartary buckwheat [10,26], suggesting a strong influence of geographical origin on genetic clustering. Based on phenotypic data (BNMS, NNMS, SD, and PH), the 313 germplasm accessions were classified into four groups, showing a difference with respect to the SNP-based clustering results (Figure 4). Plant phenotypes are influenced to some extent by environmental factors [37], while molecular markers such as SNP can reveal genetic variation at the DNA level [38]. Reliance on a single type of data may inadequately represent germplasm diversity [39]. Therefore, the discrepancy between molecular-marker-based and phenotypic clustering was anticipated, given the limited number of phenotypic traits analyzed (*n* = 4). It is well established that environmental factors exert a decisive influence on the expression of morphological and physiological traits in plants [37]. When comparing phenotypic and genetic clustering, the power to capture multidimensional phenotypic variation depends critically on the number and diversity of traits measured. A small trait set, such as that used in this study, has inherently limited ability to capture the full phenotypic space of the germplasm. From a genetic perspective, SNP-based markers can resolve fine-scale genetic differentiation independent of environmental effects, thereby providing a more stable basis for germplasm classification. Given these considerations, the integration of genetic and phenotypic data—even with a limited phenotypic dataset—contributes to a more comprehensive core collection by ensuring both allelic diversity and phenotypic representativeness. Future studies should expand phenotypic characterization to include phenological, reproductive, yield component, and seed quality traits, as yield-related traits were not investigated in this study.

The discrepancy between molecular and phenotypic clustering further underscores the necessity of integrating both data types in core collection construction. A core collection is a strategically selected subset from a large germplasm repository, designed to maintain maximum genetic diversity with minimal redundancy [40]. Yunnan Province harbors abundant Tartary buckwheat germplasm resources. Therefore, establishing a core collection in this region would facilitate their effective conservation and utilization. Core collections have been successfully constructed for various plants, such as golden camellia [41], tea plants [30], wheat landraces [32], and radish [42]. However, most studies primarily focus on constructing core collections using genetic diversity while often neglecting phenotypic variation. It is increasingly acknowledged that integrating genetic diversity with morphological and physiological traits is key to developing more precise core collections [43,44]. Phenotypic traits such as BNMS, NNMS, SD, and PH [26] are important characteristics of Tartary buckwheat. These traits vary significantly across accessions and are shaped by both genetic and environmental factors. The SNP-based subset comprised 30% of the original collection, whereas the phenotype-based subset accounted for 5%. The final core collection (Core-Merge) was obtained by merging these two subsets and removing redundant accessions, resulting in a representative and non-redundant germplasm set. Future efforts should focus on validating the agronomic performance and breeding utility of this core collection while expanding its genetic and phenotypic representativeness through the incorporation of more diverse germplasm and key adaptive traits. Notably, Core Hunter 3 has been widely used for core collection construction due to its ability to efficiently optimize genetic diversity using flexible objective functions, making it particularly suitable for integrating both molecular and phenotypic data.

## 4. Materials and Methods

### 4.1. Plant Materials

**Figure 8:**
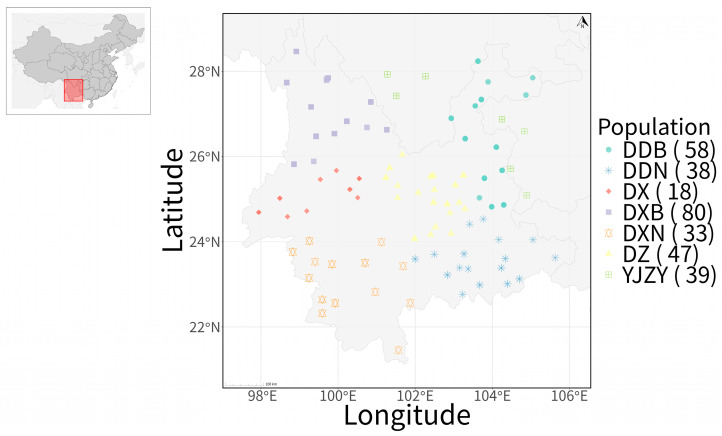
Geographic sampling of Tartary buckwheat populations in the study region Yunnan. Population codes: DDB, northeastern Yunnan; DDN, southeastern Yunnan; DX, western Yunnan; DXB, northwestern Yunnan; DXN, southwestern Yunnan; DZ, central Yunnan; YJZY, accessions introduced from Sichuan and Guizhou provinces. Note: Multiple accessions may have been collected from the same geographic coordinates or nearby locations within a population.

In this study, a total of 313 Tartary buckwheat accessions were used. All materials were collected from Yunnan, Guizhou, and Sichuan Provinces, China (Figure 8), and stored in the Yunnan Provincial Repository for Crop Germplasm Resources (Kunming, Yunnan). Detailed information on these samples, including registration codes, provinces, latitudes, longitudes, and populations, is presented in Supplementary Table S9. The field trials for phenotypic trait measurement were conducted at two locations to account for environmental variation and allow us to obtain more reliable average phenotypic values for each accession. The first site was the scientific research experimental base of the Yunnan Academy of Agricultural Sciences in Xiaojie Town, Songming County, Kunming City, Yunnan Province (25°21′23″ N, 103°06′39″ E), where planting was carried out in 2024. The second site was in Jiache Town, Huize County, Qujing City, Yunnan Province (25°59′17″ N, 103°24′44″ E), where planting was carried out in 2025. A total of 313 Tartary buckwheat germplasm accessions were planted. Each accession was planted in a single row plot that was 2 m long, with a plant spacing of 15 cm and a row spacing of 20 cm. Standard agronomic management practices, including identical irrigation and fertilization regimes, were employed. In each plot, five individual plants per accession were randomly selected at maturity for measurement, and the average values of these five plants were used to represent the phenotypic performance of each accession within each biological replicate. The experiment was conducted with three biological replicates. Healthy young leaves were collected in 2024 at the Songming site before the budding stage, immediately frozen in liquid nitrogen, and stored at −80 °C for future use.

### 4.2. Phenotypic-Trait Measurement

The evaluated traits included the number of branches on the main stem ((BNMS), i.e., the total number of primary branches originating from the main stem; (units: counts)), the number of nodes on the main stem ((NNMS) i.e., the total number of nodes from the base to the apex (unit: node)), stem diameter ((SD) measured at the midpoint between the first and second internodes (unit: mm)), and plant height ((PH), the distance from the base of the stem to the tip of the longest branch (unit: cm)). The field trial was conducted using a randomized complete block design (RCBD).

### 4.3. DNA Extraction and Whole-Genome Resequencing

Genomic DNA was extracted from 313 Tartary buckwheat germplasm leaves using a NucleoMag plant DNA extraction kit (Takara Bio Inc., Dalian, China), following the manufacturer’s recommendations. Three replicates were used for each germplasm. DNA was quantified using a NanoDrop Microvolume spectrophotometer (NanoDrop 8000, Thermo Fisher Scientific, Waltham, MA, USA), and sample integrity was evaluated using 1% agarose gel electrophoresis. Qualified DNA samples were enzymatically fragmented using MGIEasy Fast Enzyme Digestion Library Preparation Reagent Set V2.0 (MGI, Shenzhen, China), and the length of the inserted fragment was approximately 350 bp. Paired-end sequencing libraries (PE150) were prepared through a series of steps including adapter ligation, PCR amplification, purification, quality control, homogenization, circularization, exonuclease digestion, and final purification. Subsequently, the library concentration was diluted to 1 ng/μL using Qubit 2.0, and the Bioanalyzer 2100 system (Agilent Technologies, Santa Clara, CA, USA) was used to detect the size of the inserted fragments in the library. Subsequently, sequencing was performed on an MGI DNBSEQ-T7 platform by Huazhi Biotechnology Co., Ltd. (Changsha, China).

### 4.4. Read Alignment and SNP Calling

Raw sequencing reads were processed using fastp (v0.23.2) [45] to remove adapter sequences, low-quality reads, and reads with excessive N content. The filtered clean reads were aligned to the Tartary buckwheat reference genome (https://www.ncbi.nlm.nih.gov/datasets/genome/GCA_002319775.1) (accessed on 19 March 2025) [25] using Sentieon (v202212.01), which employs the BWA-MEM alignment algorithm. We also used Sentieon for joint calling, conducting joint analysis of all samples’ gVCFs to obtain the variant results for each sample. The sequencing depth and coverage compared to the reference genome were calculated based on the alignment results using SAMtools (v1.17) [46].

Based on the results of a comparison between the sample and reference genomes, SNP and InDel detection were performed using the Haplotypecall module of GATK (v4.4.0.0) [47]. SNPs were filtered based on a quality score of ≥20, a QD (variant confidence/quality by depth) < 2.0, an MQ (RMS mapping quality) < 40.0, an FS (Phred-scaled *p* value, using Fisher’s exact test to detect strand bias) > 60.0, an SOR (the symmetric odds ratio of a 2 × 2 contingency table used to detect strand bias) > 4.0, an MQRankSum (the Z score from a Wilcoxon rank sum test of Alt vs. Ref read-mapping qualities) < 12.5, a ReadPosRankSum (the Z score from a Wilcoxon rank sum test of Alt vs. Ref read position bias) < −8.0 [48], or an MAF (minor allele frequency) ≥0.1 [49]. These steps ensured the generation of a high-confidence SNP dataset for downstream population structure and diversity analyses.

### 4.5. Analysis of Population Structure and Genetic Diversity Parameters

Linkage disequilibrium (LD) pruning was performed using PLINK v1.90 with the parameter “--indep-pairwise 50 10 0.2” to choose a set of approximately independent SNP markers for population structure analysis [49]. We then applied ADMIXTURE v1.3.0 to the LD-pruned SNPs to assess population structure, selecting the optimal K value (ranging from 2 to 10) based on the lowest cross-validation error [50]. A neighbor-joining (NJ) phylogenetic tree was constructed for the 313 individuals using an identity-by-state (IBS) distance matrix and visualized in iTOL (https://itol.embl.de/) (accessed on 19 March 2025) [51]. Principal component analysis (PCA) was performed using PLINK v1.90 [52]. For phenotypic traits (BNMS, NNMS, SD, and PH), hierarchical clustering was conducted using the “cluster” package in R (v4.4.1) [53]. Using these SNPs, we calculated genetic diversity parameters, including observed heterozygosity (Ho), expected heterozygosity (H_E_), MAF, the inbreeding coefficient (FIS), nucleotide diversity (π), and polymorphism information content (PIC) via PLINK v1.90, VCFtools v0.1.16, and in-house shell scripts [54,55]. Pairwise Fst values between populations were estimated with VCFtools v0.1.16 in 500 kb non-overlapping windows across the genome [56]. Gene flow (Nm) between populations was derived from Fst using the equation Nm = (1 − Fst)/(4 × Fst). Descriptive statistics for phenotypic traits (BNMS, NNMS, SD, and PH), including maximum, minimum, mean, standard deviation, range, and coefficient of variation, were obtained using base R functions. The Sankey diagram was constructed using the ‘ggalluvial’ package (v0.12.5) in R (v4.4.1). The Shannon index was used to compute the genetic diversity index H′.

### 4.6. Construction of Core Collections

Core germplasm subsets were extracted from the 313 accessions based on SNP markers in gradient proportions of 0.05, 0.10, 0.15, 0.20, 0.25 and 0.30 [36]. Core subset selection was performed using Core Hunter 3 software, which applies advanced search algorithms (including mixed replica search and local optimization) to maximize the genetic diversity and representativeness of the selected subset. Through comparison of genetic diversity parameters, the subset with the highest representativeness and greatest genetic diversity was designated as Core1. Additionally, a candidate subset (Core2) was constructed using phenotypic data from the 313 accessions. From 313 Tartary buckwheat germplasm resources, four phenotypic traits were analyzed to establish Core2, a candidate core collection. The employed strategy was designed to minimize the Mean Difference (MD) and Variance Difference Percentage (VD) while maximizing the Coincidence Rate of Range (CR) and Variable Rate of Coefficient of Variation (VR), thus optimizing the representation of the original germplasm’s diversity. Integrating the results of genetic and phenotypic analyses, the final core collection exhibited high genetic diversity and accurately captured the range of phenotypic variation.

Genotypic and phenotypic datasets were analyzed independently to generate Core1 and Core2, respectively. The final core collection (Core-Merge) was obtained by merging the two subsets and removing redundant accessions. The construction approach focused on reducing the MD, VD, CR, and VR to effectively capture the diversity of the original germplasm [57]. Violin plots were created using the ggplot2 package in R (v4.4.1). Differences among Core1, Core-Merge, and the original collections were assessed with ANOVA followed by Tukey’s post hoc test, which was conducted through the “rstatix” package [58]. Custom functions converted *p*-values into significance notations (N.S. for non-significant, * for *p* ≤ 0.05, ** for *p* ≤ 0.01, and *** for *p* ≤ 0.001), which were then added to the plots using the geom_text() function in ggplot2. PCoA was conducted on both the original and core germplasms using the ‘ape’ package (v5.8) in R (v4.4.1), based on a genetic distance matrix calculated from SNP genotype data, to assess sample distribution patterns. The kinship matrix for Core-Merge was calculated in R (v4.4.1) using the cor function based on genotype correlation and visualized with the “ggplot2” and “pheatmap”packages.

## 5. Conclusions

In this study, we integrated SNP markers and phenotypic data to assess the genetic diversity and population structure of Tartary buckwheat germplasm. Based on an initial set of 313 accessions from Southwest China, a core collection of 105 accessions was constructed. This core collection captures the molecular, phenotypic, population, and regional diversity present in the original germplasm, providing a representative subset that preserves the genetic breadth of the original collection. A more comprehensive phenotypic characterization would further strengthen the representativeness of the core collection in future studies. The findings offer valuable insights for the conservation of, genetic research into, and breeding of Tartary buckwheat germplasm resources.

## Figures and Tables

**Table 1:** Genetic diversity analysis of different local population germplasm accessions.

District	Num SNPs	H_E_	Ho	π	MAF	FIS	PIC
DDB	1,103,513	0.3165	0.2896	3.1926 × 10^−3^	0.2378	0.0850	0.2578
DDN	1,084,913	0.3029	0.2916	3.0699 × 10^−3^	0.2268	0.0372	0.2515
DX	1,036,296	0.3266	0.2970	3.2152 × 10^−3^	0.2454	0.0907	0.2696
DXB	1,113,622	0.3277	0.2982	3.2973 × 10^−3^	0.2476	0.0899	0.2636
DXN	1,097,626	0.3068	0.2919	3.1158 × 10^−3^	0.2297	0.0485	0.2517
DZ	1,111,616	0.3235	0.2957	3.2698 × 10^−3^	0.2433	0.0859	0.2614
YJZY	1,097,524	0.3187	0.2901	3.2286 × 10^−3^	0.2399	0.0898	0.2607
ALL	1,092,159	0.3175	0.2935	3.1984 × 10^−3^	0.2387	0.0753	0.2595

Note: DDB, the northeastern region of Yunnan Province; DDN, the southeastern region of Yunnan Province; DX, the western region of Yunnan Province; DXB, the northwestern region of Yunnan Province; DXN, the southwestern region of Yunnan Province; DZ, the central region of Yunnan Province; YJZY, the resources that have been introduced from Sichuan and Guizhou. Num SNPs, number of single-nucleotide polymorphisms (SNPs); H_E_, expected heterozygosity; Ho, observed heterozygosity; π, nucleotide diversity; MAF, major allele frequency; FIS, inbreeding coefficient; PIC, polymorphic information content.

**Table 2:** Genetic differentiation coefficient and gene flow between different Tartary buckwheat populations.

District	DDB	DDN	DX	DXB	DXN	DZ	YJZY
DDB		8.2234	22.0158	22.2078	8.8634	49.9095	37.3643
DDN	0.0295		7.1209	6.4762	13.2994	10.3743	5.2287
DX	0.0112	0.0339		35.7264	14.5010	−476.6855	17.6686
DXB	0.0111	0.0372	0.0069		8.8449	31.4413	27.2503
DXN	0.0274	0.0185	0.0169	0.0275		14.1418	6.6477
DZ	0.0050	0.0235	−0.0005	0.0079	0.0174		24.3490
YJZY	0.0066	0.0456	0.0140	0.0091	0.0362	0.0102	

Note: Fst values (lower diagonal) and Nm values (upper diagonal) between pairwise populations. Fst values of 0–0.05, 0.05–0.15, and 0.15–0.25 indicate low, moderate, and high genetic differentiation, respectively. Nm values > 4 indicate high levels of genetic exchange; Nm values < 1 indicate low gene flow.

**Table 3:** Statistical analysis of phenotypic traits in Tartary buckwheat germplasm.

Traits	Mean	Max	Min	Range	SD	CV	H′
BNMS	6.8	24.3	4.0	20.3	1.5	21.3	0.8
NNMS	19.1	28.0	14.8	13.2	2.0	10.3	1.6
SD (mm)	9.8	13.1	7.6	5.5	0.8	8.5	1.8
PH (cm)	151.3	209.4	121.8	87.6	12.2	8.1	1.7

Note: Mean, average; Max, maximum; Min, minimum; SD, standard deviation; CV%, coefficient of variation; H′, Shannon’s information index; BNMS, number of branches on the main stem; NNMS, number of nodes on the main stem; SD, stem diameter; PH, plant height.

**Table 4:** Comparison of genetic diversity among 313 germplasm samples at different sampling proportions.

Candidate Set	Ho	H_E_	MAF	FIS	π	PIC
5%	0.5841	0.5841	0.3132	−0.4954	0.3371	0.3074
10%	0.5919	0.5919	0.3168	−0.4915	0.3331	0.3125
15%	0.5905	0.5905	0.3159	−0.4923	0.3333	0.3116
20%	0.5913	0.5913	0.3170	−0.4880	0.3323	0.3129
25%	0.5902	0.5902	0.3162	−0.4896	0.3319	0.3121
30%	0.5928	0.5928	0.3174	−0.4908	0.3330	0.3131
100%	0.5941	0.5941	0.3181	−0.4916	0.3319	0.3136

Note: H_E_, expected heterozygosity; Ho, observed heterozygosity; π, nucleotide diversity; MAF, major allele frequency; FIS, inbreeding coefficient; PIC, polymorphic information content.

## Data Availability

The raw sequencing data have been deposited in the National Genomics Data Center (NGDC: https://ngdc.cncb.ac.cn/) (accessed on 12 March 2025) Genome Sequence Archive (GSA), under BioProject accession number PRJCA058783. Data sharing is available upon request.

## Supplementary Materials

The following supporting information can be downloaded at https://www.mdpi.com/article/10.3390/plants15081197/s1, Table S1: Statistics of data quality control results; Table S2: Mean sequencing depth and coverage of individuals; Table S3: Statistics of chromosome-related variation information; Table S4: Statistics of count, frequency and percentage of transition types; Table S5: Ancestry proportions (Q values) of 313 accessions for four ancestral groups (K = 4) inferred by ADMIXTURE; Table S6: Average value of phenotypic data; Table S7: Core germplasm; Table S8: Construction of core germplasm based on phenotypic traits; Table S9: Registration code, Province, latitude, longitude, and population data.
